# Teaching medical students hematopathology: a randomized crossover study comparing direct inspection by light microscope versus projected images

**DOI:** 10.3389/fmed.2024.1340359

**Published:** 2024-08-27

**Authors:** Sultan Alqahtani, Sami Al-Nasser, Sajida Agha, Mohamud S. Mohamud

**Affiliations:** ^1^Department of Basic Medical Sciences, College of Medicine, King Saud bin Abdulaziz University for Health Sciences, Riyadh, Saudi Arabia; ^2^King Abdullah International Medical Research Center (KAIMRC), Riyadh, Saudi Arabia; ^3^Department of Medical Education, College of Medicine, King Saud bin Abdulaziz University for Health Sciences, Riyadh, Saudi Arabia; ^4^Department of Primary Care and Public Health, School of Medicine, Imperial College London, London, United Kingdom

**Keywords:** hematopathology, projection method, light microscopy, medical education, randomized crossover

## Abstract

**Background:**

Students’ ability to diagnose various blood disorders could be substantially improved by continuously reviewing approaches toward teaching hematology. This study aims to compare the effectiveness of light microscopes and projected images on students’ learning and determine medical students’ perception of these teaching methods.

**Methods:**

A randomized trial was conducted using a crossover design. Two groups, each with 30 students, were subjected to teaching methods based on light microscopes and projected images alternatively.

**Results:**

No differences were found in the two study groups’ baseline characteristics, such as median age, sex, and prior academic performance, as well as in the pre-test scores. Post-test scores were significantly higher among students subjected to the projection method than in the control group (Mean ± SD = 9.8 ± 1.7 vs. 5.1 ± 1.3, *p* < 0.001). In the post-cross-over assessment, 85% (*n* = 51) of students reported their satisfaction for the projected images, and 78% (*n* = 47) of students were willing to be taught by projection. Students perceived that the projection method facilitated participation and better involvement in discussions, improved learning, provided greater motivation, and eventually increased comprehension and efficiency.

**Conclusion:**

The projection-based teaching method is more effective in improving knowledge and achieving intended learning outcomes. Students tend to prefer the projection method over the laboratory-based method and perceive it as an effective method to enhance their learning of hematology.

## 1 Introduction

Hematopathology is the study of blood disorders as well as hematologic malignancies, or tumors that affect bone marrow and blood cells. These include lymphomas, leukemias, and other disorders of the bone marrow and blood. Students’ ability to diagnose various blood disorders could be substantially improved by continuously reviewing the approaches toward teaching hematology at the undergraduate level (1). Several researchers have compared the efficiencies of teaching strategies or approaches based on virtual and traditional light microscopies; however, they have not found any difference between the projection approach and the different educational strategies on student learning (2–4). According to Broudy and Hickman, laboratory-based microscopy teaching in hematology is not a popular teaching strategy compared to projection-based teaching, which is extensively utilized (5). Similarly, Aron et al. reported no significant difference between direct inspection by light microscope and projected images for teaching hematopathology to medical students (6). However, a study indicated that virtual microscopy is a useful educational tool and that students prefer using it while learning to evaluate images of clinical specimens (7).

Moreover, Broudy and Hickman in their research further revealed that although laboratory skills (20%) and microscopy (60%) were used less frequently by course directors, projection-based learning was utilized by them extensively (5). Following the introduction of virtual microscopy, a number of studies (8, 9) have examined the educational benefits of the technology and also contrasted its performance with traditional light microscopy, but not with image projection.

Teaching staff working in the hematology section of the King Saud bin Abdulaziz University for Health Science’s (KSAU-HS) College of Medicine believes in the continuous quality improvement of medical education and hence applies various teaching approaches during clinical and laboratory academic sessions. Hematology, which is taught in the third year of the M.B.B.S. with a hybrid curriculum, is mainly grounded in conventional lectures and person-centered learning approaches, such as basic clinical science (practical sessions) and problem-based learning. Light microscopy is mostly utilized in the hematology section for investigating hematological conditions. During hematopathology, academic-session light microscopy is used to examine bone marrow slides and peripheral blood smears using specific instructions. In particular, each practical/laboratory session is conducted for 3–4 h so that students can comprehend and perform microscopy and interpret their findings.

Instructors or faculty mostly lead students during the practical sessions in the laboratory so that they may learn and discuss various aspects of bone marrow images and peripheral blood smear. One instructor can teach approximately 10 students at a time. A considerable downside of this practical approach is that it calls for the recruitment of staff and sufficient space for the students (5). Improved learning outcomes can be achieved through optimization of working memory, which can be done by practice and minimization of extra cognitive load. Various teaching strategies can be applied for minimizing extra cognitive load and increasing the germane cognitive load at the same time (10, 11).

Clinical reasoning and the active learning process can be greatly improved through using computer-based and conventional techniques (9). While teaching undergraduate medical students, one can utilize virtual microscopy and projected-image techniques in place of laboratory-based sessions (1). However, projected images are not fully utilized in most institutions in Saudi Arabia and particularly in the College of Medicine of KSAU-HS. In order to investigate the similarity of educational outcomes following direct inspection by light microscope versus projected images for hematopathology education in Saudi Arabia, it is hypothesized that the utilization of the projection method (projected slides) would lead to significant improvements in diagnostic efficiency compared to the utilization of light microscopy (a laboratory method) for the same purpose as it allows the students’ more active participation. This study aimed to determine and compare the effectiveness of the two teaching methods: direct examination through light microscopy and projected images. It also aimed to assess the medical students’ perception and knowledge of practical hematopathology using light microscopes and projected images at the College of Medicine of KSAU-HS. To determine and compare the academic outcomes of the two teaching methods—direct examination through light microscopy and projected images (photomicrographs of glass slides)—a prospective, randomized controlled trial was conducted involving third-year medical students.

## 2 Materials and methods

A randomized study was conducted using a crossover design at the College of Medicine associated with KSAU-HS to compare the effectiveness of direct inspection by light microscope versus projected images as the teaching methodology for hematopathology. All students were equally exposed to both methods in this study design.

**Figure 1:**
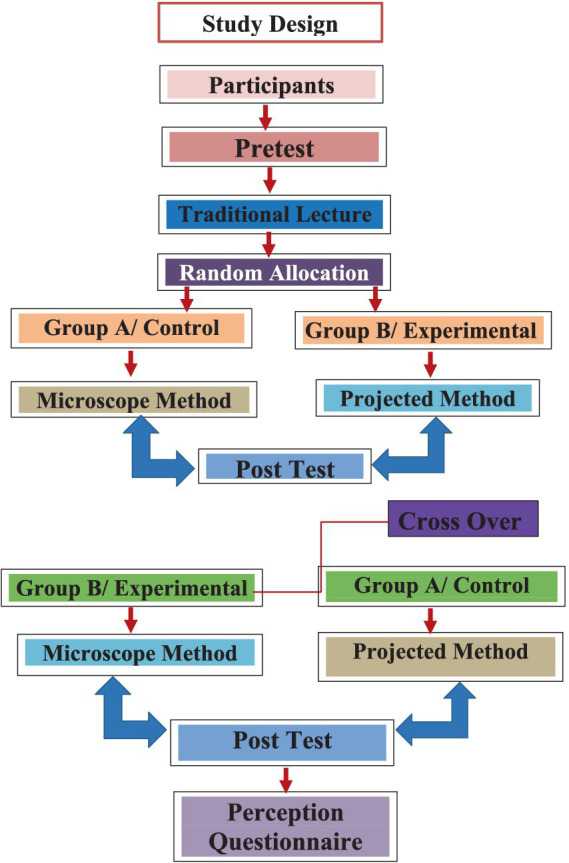
Schematic diagram illustrating randomization of two groups and study protocol.

In total, 60 third-year, male medical students were selected from the students’ enrollment list using a simple random sampling technique (Figure 1). First, a list of all the students (*N* = 60/out of 120) who volunteered to take part in the study was made. A number was assigned to each participant as an identifier. Using the random numbers that were assigned to each participant, the list of participants was sorted into ascending order. Participants were further divided into two groups by simply allocating individuals to each group alternately, beginning at the top of the list. Authors explained the purpose of the study and each participant’s role during that process.

Initially, a pre-test was conducted with all 60 students using a structured multiple type questions to assess their baseline knowledge of hematopathology and ability to recognize the hematological morphology, which was followed by a didactic lecture on the same subject. Later, all study participants were randomly allocated to group A (the microscopic method/control group) or group B (projected method/experimental group), with 30 participants in each group. Both groups were delivered short instructional sessions simultaneously. On the day one, those assigned to group A, proceeded to the hematopathology laboratory where case discussions utilizing direct inspection (a laboratory method) were presented. Group B on the other hand, remained in the classroom where case discussions utilizing projected slides (a projection method) was done.

In the microscopic group, students were instructed to review and examine the peripheral blood smears in these case studies while examining the smears on the microscope. The instructor then explained the key findings and discussed several important questions with them. On average, 10 medical students shared one light microscope at a time. Each student had adequate time (Total 20 min; 2 min each student) during his turn to examine the smears under the microscope.

In the experimental group, however, high-definition photographs of peripheral blood smears relating to the same case studies were shown for 2 min each to the students and then discussed in the classroom. After that, they discussed the same questions as done in the microscopic method. All of the slides and images used in the hematology course were created by college of medicine professionals and used in the current study with permission from the relevant faculty.

After the teaching session, students in the experimental and control groups were asked to identify and diagnose six case studies of various kinds of leukemia and anemia within the syllabus taught in the didactic lecture.

On day two, after attending the lecture on hematology related topics such as sickle anemia, the study participants were instructed to swap groups; group A students were subjected to projected images, and group B students were subjected to the microscopy-based approach as teaching methodology. The same instructors were retained for each method. After the successful completion of the teaching session on both days, each group was offered a post-test examination comprising 12 open-ended vignette-based questions to measure learning outcomes by assessing the students’ knowledge and diagnostic ability based on the morphologic recognition of hematopathology, clinical laboratory finding, diagnosis, and management. The two post-tests were identical in both days.

After the crossover academic sessions and the post-tests by each group, a self-administered, semi-structured questionnaire was administered to determine which of the two methods the students preferred and their level of satisfaction toward the two teaching methods using a 5-point Likert scale (1 = *strongly disagree*, 5 = *strongly agree*). Students were also asked to provide suggestions about if these methods could be improved. The validation was done by the content experts.

All the collected data were recorded on an Excel sheet and analyzed using SPSS (version 21, IBM). Descriptive statistics were calculated for the intervention and control groups as mean, median, standard deviation, and proportions. A Chi-square test was performed to identify any statistically significant differences in the intervention and control groups. To compare the effectiveness of the teaching methodologies implemented in the intervention and control groups, the independent samples t-test was used. A *p*-value equal to or less than 0.05 was considered statistically significant. The study was conducted in accordance with the Declaration of Helsinki and approved by the Institutional Review Board of King Abdullah International Medical Research Centre (IRB #SP19/309/R).

## 3 Results

**Figure 2:**
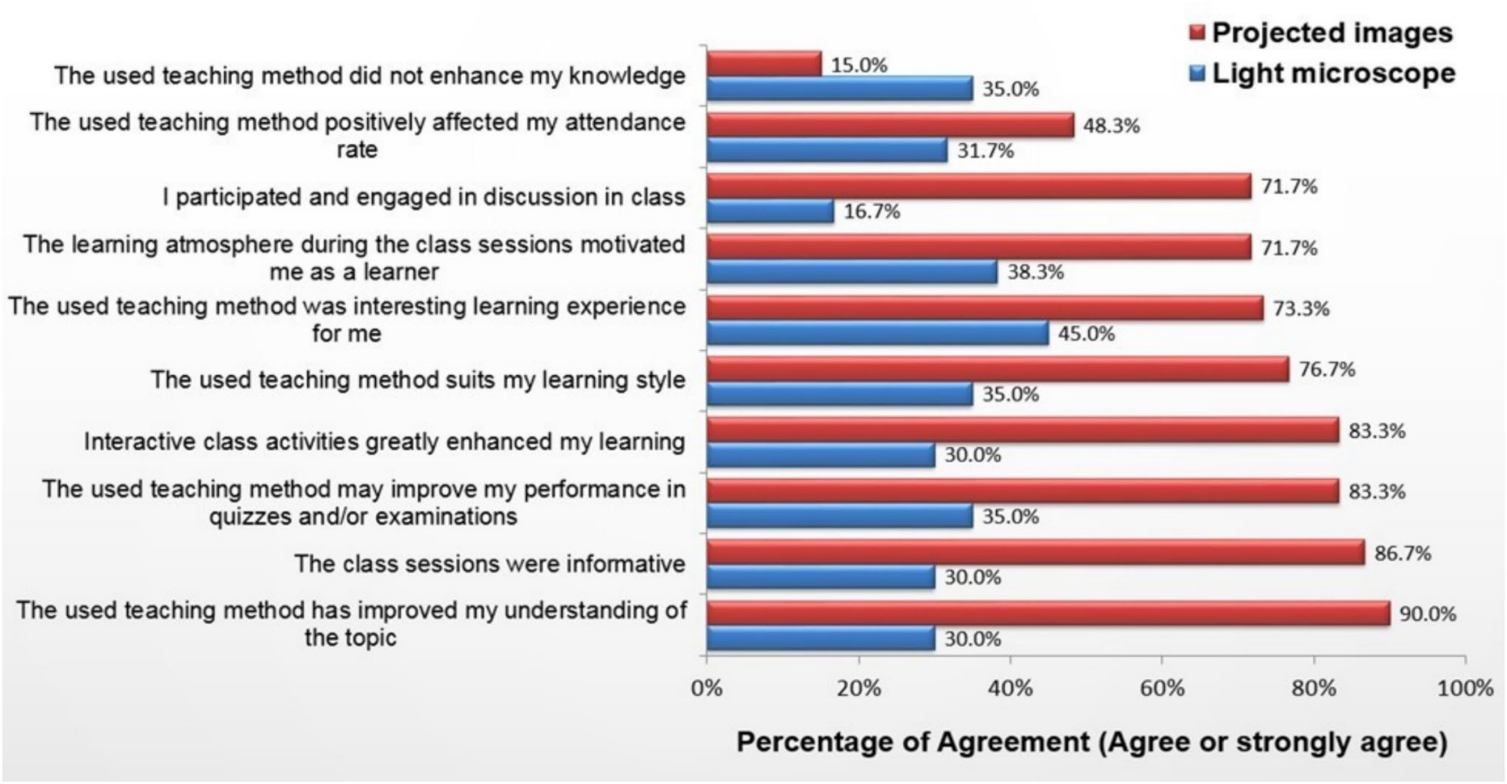
Students’ perception of light microscopy and projected images.

**Figure 3:**
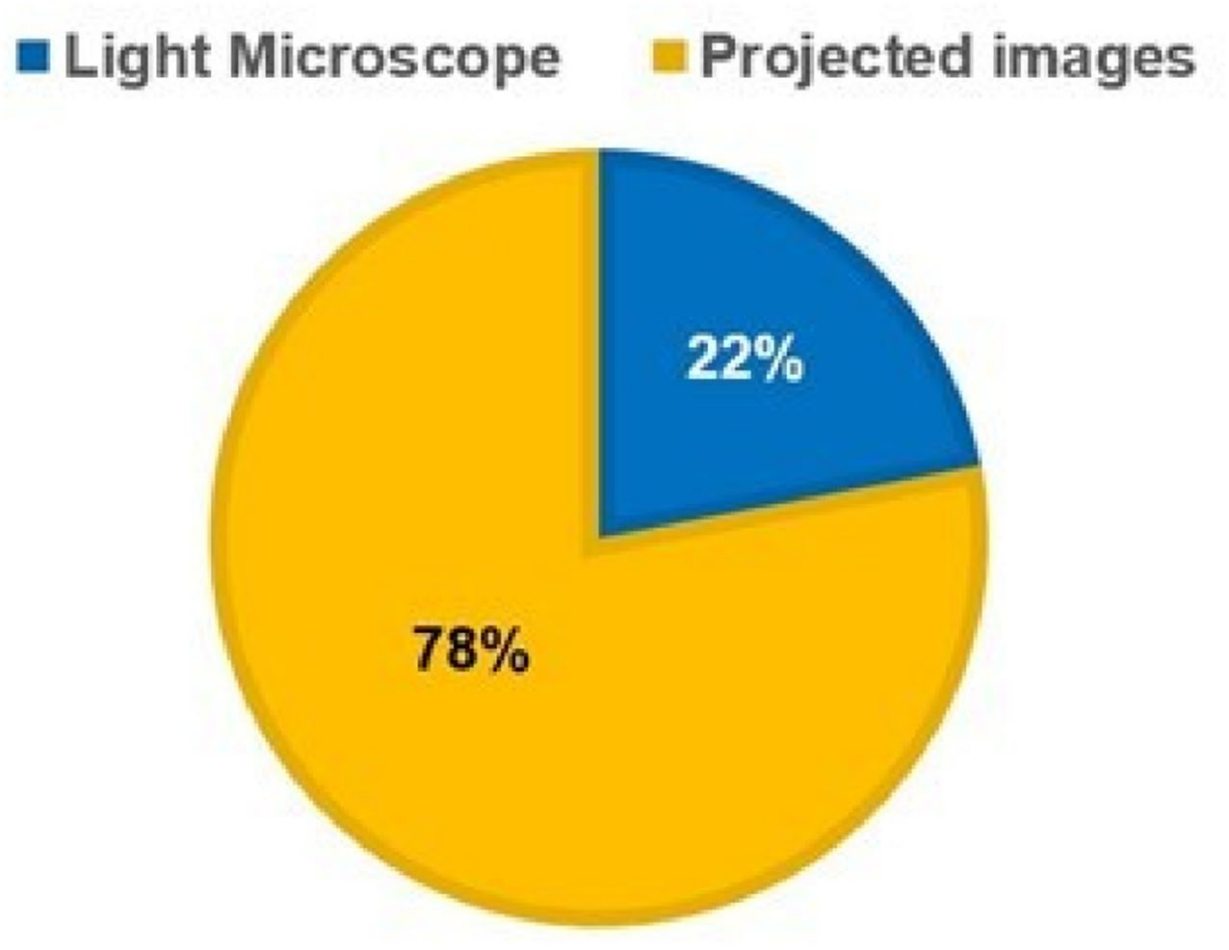
Students’ preference of light microscope vs projected images.

This study involved 60 third-year, male medical students. Figures 2, 3 show the students’ preferences and perceptions. At baseline, no significant differences were observed in the median age and cumulative grade point average or academic performance of students in the intervention or control groups (Table 1). Similarly, no significant differences were observed in the pre-test scores of the two groups; however, the post-test scores were found to be significantly higher in group B (intervention group; Mean ± SD = 9.8 ± 1.7) compared to group A (control group; Mean ± SD = 5.1 ± 1.3), using the independent samples t-test with a *p*-value <0.001 (Table 2).

**Table 1:** Comparison of students’ demographic factors.

Factor	Group					
		Light microscope (*N* = 30)		Projected images (*N* = 30)		*p*-value
		*N*	%	*N*	%	
Age (years)	19–20	18	60	21	70	0.77
	21+	12	40.0	9	30.0	
cGPA	3.51–4.50	11	36.6	13	43.3	0.89
	4.51–5.00	19	63.3	17	56.7	

cGPA, cumulative grade point average.

**Table 2:** Comparison of two groups’ mean test scores.

Score	Groups	Mean ± SD	*p*-value
Pre-score/10	Light microscope (A) (*N* = 30)	4.5 ± 1.5	0.61
	Projected images (B) (*N* = 30)	4.7 ± 1.6	
Post-score/10	Light microscope (A) (*N* = 30)	5.1 ± 1.3	< 0.001
	Projected images (B) (*N* = 30)	9.8 ± 1.7	

Among students, 85% (*n* = 51) reported their satisfaction with the projected-images method (Figure 2), and 78% (*n* = 47) indicated that they would be willing to learn using the projection method. However, 22% (*n* = 13) preferred the microscopy-based teaching approach when they were made to select one from the two teaching approaches (Figure 3), and 54% (*n* = 33) reported that the lack of space and instruments in the laboratory hindered their learning in the laboratory-based method.

Most students favored the projected-images approach. Students perceived that the projection method facilitated engagement and better involvement in discussions, improved learning, provided greater motivation, and eventually increased comprehension and efficiency.

## 4 Discussion

Modern technological advancements have not only revolutionized health, engineering, and other fields, but have also made revolutionary changes in the field of education. The scientific revolution in education has particularly affected medical education, where projected images and virtual microscopy have become a common resource to facilitate enhanced learning among undergraduate medical students. The use of digital technology is already in progress at many reputed medical schools to teach several organ-specific courses such as hematology, anatomy, and histology, hence reducing laboratory sessions (12).

The direct examination of glass slides serves as the gold standard for the conventional education of medical students and residents. Considerable benefits are offered by teaching with conventional microscopy; for instance, the preparation of glass slides proves to be economical, and students gain expertise in microscopic examination. Students of microbiology, pathology, and histology are taught with conventional microscopy during their initial stages of education. Yet, light microscopy also has certain downsides: microscopes are costly instruments calling for maintenance and storage, and in cases where slides are utilized by a high number of students, slide collections are quite difficult to obtain and maintain (4).

The two teaching methods studied in this research differed in several ways. The laboratory-based teaching method calls for a high level of active involvement of students in observing the slides and interpreting the findings. Conversely, the projection method does not require students to participate in the direct observation of slides and processing of data. Nevertheless, both teaching methods involve discussions between students and instructors about the case study. It has been established that active learning is more advantageous for retention and learning and is also well supported by many advocates as a learning method for histology and microscopic anatomy (13–15).

In this research, the projection method was found to be more effective since the scores in testing performance of students subjected to the projection method were significantly higher than those of the students subjected to the laboratory-based method. This is because the projection method offers convenient examination of slides, observation of images with clarity, greater accessibility allowing independent as well as group study, efficient consumption of time, and optimal learning leading to students’ better performance. In addition, most students preferred to be taught with the projection method. This can be explained by the perceived reasons mentioned by the students, such as better group interaction and discussions, enhanced understanding, and increased motivation owing to overall improved learning experience. However, 22% of the students still preferred laboratory-based teaching probably because of their preference for traditional approaches for various reasons or individuals’ inability to understand and work in a group (16, 17). A multicenter study identified that medical students with specific learning styles tend to favor active learning, conceptual methods, analytical strategies, and independent learning as compared to group sessions (16). In another study, the learning styles of first-year medical students belonging to an institute were investigated through the Visual, Auditory, Reading/writing, Kinesthetic Questionnaire. The students’ responses indicated that most learners tend to like multiple learning styles, and most of them opt for a combination of 3–4 learning methods (17). During this research, only 5.4% of students preferred to be purely visual learners and stressed the significance of utilization of several learning strategies and inclusion of image-based coursework.

However, limited resources in the laboratory, such as a lack of space and equipment for examining slides, can be a reason for the students’ support of the projection method. Conversely, the analysis of images presented through PowerPoint in huge classrooms proved to be quite convenient. Such support for digital approaches has been reported by several studies comparing conventional microscopy with the latest digital methods. As per these reports, digital approaches offer greater accessibility and are more efficient (3, 18). Conventional approaches can be improved through increasing space in the laboratory, providing more equipment, and hiring more instructors; however, this calls for substantial investment. Several medical schools have started utilizing virtual slide collections for strengthening histology and histopathology courses, and in certain cases, conventional light microscopy has been entirely replaced with contemporary digital approaches (19, 20). In several medical education programs, instructors and learners have highly rated the integration of virtual microscopy in educational programs (20). In some cases, learners and instructors tend to prefer digital approaches to conventional approaches (19–21), and the increased availability of computers as compared to microscopes in current educational settings might have contributed to this preference (22).

The findings of the current study align with a US-based study, which reported that the participants involved demonstrated a preference for projection methods compared with conventional light microscopy. However, this US-based research did not report any statistically significant difference in the pre-test and post-test scores of students taught with two different teaching methods (2). This indicates a possible disparity between apparent advantages or perceived benefits versus learning efficacy. Large-scale studies with sufficient sample size and repeated measures are warranted to establish the effectiveness of the selected teaching methods as the current study only involved one-time exposure to each teaching method, followed by a post-test and assessment of students’ perceptions. The laboratory-based group session as well as the projection method session was followed by a question-and-answer session. However, the particular skills of the faculty involved in implementing a specific teaching method and their personal preferences for a certain teaching method might have affected the quality and accuracy of implementation.

This study had some limitations. It was conducted only in one medical college among male, undergraduate medical students, which compromises the generalizability of the findings with regard to the effectiveness of the projection method. Because of the segregation system, female students’ perspectives were not taken into consideration, which may limit the application of the findings in other educational settings. Furthermore, this study involved one-time exposure to both techniques; however, multiple exposures to both teaching methods could be more helpful to determine the effectiveness of both methods in the long run. Additionally, students’ perceptions and learning outcomes may be impacted by using one microscope for 10 students. In order to increase student engagement, future research should give students more opportunities for hands-on learning and microscope exposure.

For future research, it is necessary to analyze integrated learning methods involving improved team-based learning methods and adapt the curriculum with the projection method and virtual microscopy by introducing a combination of the two. Long-term follow-up studies should also be carried out to evaluate how different teaching strategies affect students’ ability to retain information over time.

## 5 Conclusion

In this study, the projected-images method showed better academic outcomes than light microscopy for teaching hematopathology. Considering the significant number of students in undergraduate medical education and the lack of resources for laboratory-based learning sessions, a classroom-based strategy such as the projected-images method should be favored as it seems to be more beneficial for learning. The projected-images method uses computer systems efficiently to integrate the conventional and digital teaching methods for hematology. Integrated learning methods involving improved team-based learning options and innovative digital techniques such as virtual microscopy serve as an acceptable combination of the two teaching approaches. However, innovative teaching approaches need to be effectively analyzed before implementation to evaluate their effect on learning efficiency.

## Data Availability

The raw data supporting the conclusions of this article will be made available by the authors, without undue reservation.
