# Supplementary material for: Teaching medical students hematopathology: a randomized crossover study comparing direct inspection by light microscope versus projected images
Source: Front Med (Lausanne). 2024 Aug 27;11:1340359. doi: 10.3389/fmed.2024.1340359 (PMC11385680; doi:10.3389/fmed.2024.1340359)
Supplement: Supplementary file 1 [file Data_Sheet_1.pdf]

RYD-19-419812-100012

King Abdullah International Medical Research Center
KAIMRC
APPROVED
IRB
Institutional Review Board

Serial Number: Group: Date:

Perception of students toward the two teaching methods

This form concerns your perception regarding the used teaching method

Age:________ **Stream:** **1 [ ]** **2 [ ]**

Gender: Male Female

Please answer the following questions based on the following scale: 1=*Strongly Disagree*, 2=*Disagree*, 3=*Neutral*, 4=*Agree*, 5=*Strongly Agree*

	Traditional (Light microscope)					Projected Images				
1. The used teaching method was interesting learning experience for me	1	2	3	4	5	1	2	3	4	5
2. The used teaching method did not enhance my knowledge	1	2	3	4	5	1	2	3	4	5
3. The class sessions were informative	1	2	3	4	5	1	2	3	4	5
4. The learning atmosphere during the class sessions motivated me as a learner	1	2	3	4	5	1	2	3	4	5
5. The used teaching method has improved my understanding of the topic	1	2	3	4	5	1	2	3	4	5
6. The used teaching method positively affected my attendance rate	1	2	3	4	5	1	2	3	4	5
7. The used teaching method may improve my performance in quizzes and/or examinations	1	2	3	4	5	1	2	3	4	5
8. The used teaching method suits my learning style	1	2	3	4	5	1	2	3	4	5
9. Interactive class activities greatly enhanced my learning	1	2	3	4	5	1	2	3	4	5
10. I participated and engaged in discussion in class	1	2	3	4	5	1	2	3	4	5

Serial Number: Group: Date:

Please answer the following questions:

I. Which form of teaching do you prefer?

- o Light microscope
- o Projected images

II. What do you think about the class session (traditional (Light microscope) & Projected images)?

(Give some general thoughts – pros and cons)

-

-

-

I. What do you think about the used teaching method as a whole (traditional (Light microscope) & projected images)? (Give some general thoughts – pros and cons)

-

-
